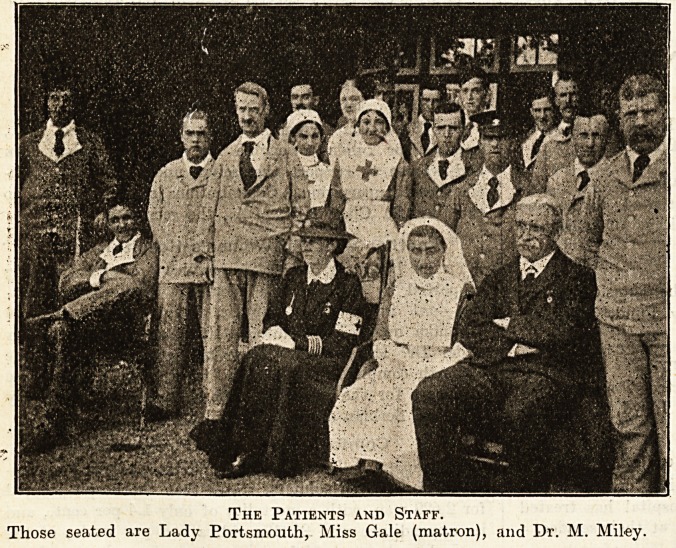# The Convalescent Soldier: Wakeswood Home, near Andover

**Published:** 1916-07-08

**Authors:** 


### THE CONVALESCENT SOLDIER.

### Wakeswood Home, near Andover.

Shortly after the outbreak of war the Countess of Portsmouth placed certain beds at her Hampshire house, Hurstbourne Park, at the disposal of the authorities for the benefit of convalescent soldiers. In April 1915, to place the work on an institutional basis, Lady Portsmouth took a commodious house known as "Wakeswood," to which the patients were transferred and where eighteen convalescents can be accommodated. "Wakeswood," the private house of Colonel Longfellow Cooper, who was wounded in Gallipoli after successfully organising the 1st Wessex Army Service Corps, stands on the sunny side of the hills which border the narrow valley that runs through Hurstbourne Priors.

### The Staff and its Voluntary Workers.

Here, for the past thirteen months, convalescent soldiers have been received from the Tidworth Military Hospital, to which it is affiliated. Colonel Faichnie, R.A.M.C., the officer commanding the latter institution, periodically pays visits of inspection, and Lady Portsmouth is often in attendance personally to supervise the working of the home. Being intended for convalescent cases only, no operations of any kind are performed, and any case which required surgical treatment would be transferred back to Tidworth at once. The medical staff therefore consists of Dr. Miles Miley, honorary medical officer, and Mr. Cardwell, visiting medical officer. The matron is Miss Gale, who was trained at Beckenham, assisted by a resident trained masseuse, Miss Long, and a resident V.A.D. nurse. In addition there are five certificated Red Cross helpers, most of whom are residents in St. Mary Bourne and neighbouring villages. Each of these undertakes a week's attendance in rotation. Their hours are from 9 to 12.30, when luncheon is provided for them in the institution, and they are generally finished by about 2 o'clock. Their work includes some of the relatively simple dressings required, and a certain number of lighter household duties. The domestic work is performed by a cook and a kitchenmaid, assisted by the patients themselves, who do all the washing-up, polish the brasses, and so on. In the pleasant garden they play bowls, croquet, quoits, and skittles.

The patients come directly from Tidworth, but indirectly from anywhere, and many branches of ordinary civilian life at one time or another have been represented. For instance, among them have been dentists, a professional dog-breeder, chauffeurs, engineers, miners, a bank clerk, a Liverpool policeman, a Canadian, and a workhouse boy who seemed to have no human belongings at all. At the moment the patients include an artist, Private Humphreys, who has made several water-colour sketches of the valley during his stay. The type of patient depends, of course, upon the regiments which happen to be stationed at Tidworth.

The House from the Garden.

The cases are both medical and surgical, recovering from accidents and operations or from influenza, bronchitis, and pneumonia. The average length of stay is three weeks or a month. One or two cases have paid much longer visits; but the term is largely in the discretion of Dr. Miley, who can advise the authorities at Tidworth of the desirability or otherwise of extending the period of convalescence.

The Patients and Staff.
Those seated are Lady Portsmouth, Miss Gale (matron), and Dr. M. Miley.

### The Wakeswood Gramophone.

The administration of convalescent patients is always something of an art, as sanatorium superintendents have long realised. But the rules at Wakeswood are simple. The patients go out in twos or threes, and provided that they are home punctually to meals, have few restrictions beyond the general military regulation which places all public-houses out of bounds, and the particular rule which limits them to the garden after tea-time. No smoking is allowed in the bedrooms or before breakfast. A certain amount of housework is performed by the men. Their principal amusement, apart from the pleasures provided by the site, the garden, and the weather, is a gramophone.

## Figures and Tables

**Figure f1:**
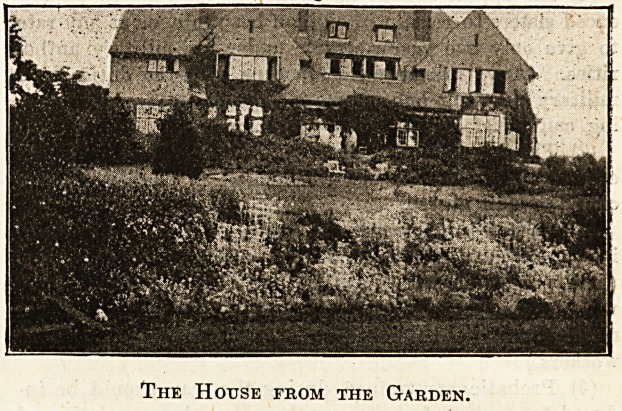


**Figure f2:**